# Development in Fiber-Reinforced Polymer Composites: 1st Edition

**DOI:** 10.3390/polym18070826

**Published:** 2026-03-28

**Authors:** Mercedes Santiago-Calvo, Juan Carlos Merino

**Affiliations:** 1 Foundation for Research and Development in Transport and Energy (FUNDACIÓN CIDAUT), Parque Tecnológico de Boecillo, 47151 Valladolid, Spain; 2 Department of Condensed Matter Physics, Escuela de Ingenierías Industriales, University of Valladolid, Paseo del Cauce, 59, 47011 Valladolid, Spain

This Special Issue entitled, “Development in Fiber-Reinforced Polymer Composites: 1st Edition”, highlights the latest advancements in fiber-reinforced polymer (FRP) composites. These composite materials, known for their exceptional strength, stiffness, and lightweight properties, are crucial in various industrial sectors such as construction, automotives, aerospace, and maritime, among others. This Special Issue emphasizes the importance of sustainable alternatives to synthetic fibers and petro-based matrices, focusing on recycling processes and the use of bio-based polymers and natural fibers. The studies on this topic focus on different aspects such as mechanical and thermal optimization, flame retardancy, and damping properties, with a special emphasis on eco-friendly solutions.

The use of fibers or reinforcements of natural origin to produce composites, as well as the use of eco-friendly matrices, is addressed in this Special Issue and in the recent literature. Park et al. [1] enhanced the stiffness and oil resistance of fluorosilicone rubber composites by reinforcing them with untreated fibrillated cellulose. The results showed significant improvements in mechanical properties, including an increased Young’s modulus and elongation at break, without compromising tensile strength. The composite systems also exhibited enhanced stress-relaxation characteristics under oil immersion, making them suitable for advanced elastomer applications. Dénes et al. [2] proposed ecological thermal insulation materials using sheep wool, with acrylic-polyurethane resin and natural rubber latex as binders. The composites developed showed thermal conductivity values between 0.0324 and 0.0436 W/mK, meeting national thermal performance criteria. Further analysis revealed good sound absorption characteristics but low resistance to microorganisms and water-related tests. Ares-Elejoste et al. [3] developed sustainable prepregs using furan resin and basalt fiber, focusing on improved fire behavior. The flame-retarded prepreg released fewer toxic gases during combustion compared to the non-flame-retarded version, although the latter resulted in lower smoke density. The flame-retarded material met the highest safety level (R1HL3) for railway vehicle interiors, indicating its suitability for such applications. Beyond the contributions collected in this Special Issue, other recent studies [4,5,6,7] have expanded the scope of natural fiber eco-based composites, covering a wide range of fiber types (jute, kenaf, banana, coir), bio-matrices, fabrication methods, and life-cycle analyses, and addressing remaining challenges such as fiber–matrix adhesion, moisture uptake, flammability, durability, and end-of-life management.

Outlined below are notable investigations into the application of recycled materials and reinforcements, as well as the recyclability of composites. Almahri et al. [8] developed a hybrid FRP composite using recycled Polyethylene terephthalate (PET) and carbon fiber (CF). They found that the composites containing 20% CF had the highest elastic modulus, increasing by 97.5% compared to that of pure PET. The optimal 20% CF/PET composite exhibited the best mechanical strength, as confirmed by tensile tests and Scanning Electron Microscopy (SEM) analysis. García-Martínez et al. [9] demonstrated the efficiency of a maleated interfacial agent, derived from atactic polypropylene industrial waste, in polypropylene/short CF composites. This investigation highlighted the significant impact of processing methods on the thermal and dynamic mechanical properties of the composites. The use of the interfacial agent resulted in a 200% increase in stiffness and a 400% improvement in viscous response, with variations depending on whether compression or injection molding was used. Butenegro et al. [10] developed new thermoplastic composites using recycled carbon fiber-reinforced polymer (CFRP) waste rods with polyamides (PA11 or PA12) as the polymeric matrix. The composites studied showed well-distributed fibers and similar mechanical properties between the two sets. The glass transition temperature (Tg) increased slightly, indicating improved thermal stability. Ferjan et al. [11] evaluated end-of-life (EoL) technologies for biocomposite recycling in the aviation industry using a structured, five-step approach. They identified solvolysis and pyrolysis as the top two viable EoL recycling technologies based on life-cycle assessment (LCA) and techno-economic analysis (TEA). These methods were found to be technically, economically, and environmentally sustainable for treating biocomposite waste. In addition to the investigations presented in this Special Issue, several recent studies have further explored the use of recycled materials and the recyclability of composites [12,13,14]. These studies have broadened the field—encompassing high-performance thermoplastic matrices with recycled carbon fiber, systematic reviews of long-fiber reuse from waste composites, and life-cycle/techno-economic valorization of glass-fiber composite waste streams. These studies address key challenges such as maintaining mechanical performance after recycling, enabling closed-loop reuse of reinforcements, and ensuring environmental and economic viability of recycling processes.

Several of the manuscripts published in this Special Issue cover aspects related to the optimization of the manufacturing process for FRP composites. Tipboonsri et al. [15] optimized the thermoplastic pultrusion parameters for jute and glass fiber-reinforced polypropylene composites. The results showed that increasing the molding temperature initially improved the composites’ mechanical properties but led to a decrease beyond a certain point due to increased void content and fiber degradation. Higher pulling speeds resulted in poorer mechanical properties due to insufficient resin impregnation and increased void content. Chandra Chaparala et al. [16] examined the impact of die temperature on the resin cure state in the pultrusion process for FRP composites based on vinyl ester resin. Higher die temperatures increased the degree of cure, with a curing rate of 97.7% achieved at 140 °C. However, the best mechanical performance was observed at 120 °C due to lower internal stress. These findings will help optimize die temperatures for better thermal and mechanical properties in pultruded composites. Bittrich et al. [17] investigated the resin transfer molding (RTM) infiltration of fiber-reinforced composites made using tailored fiber placement. The results showed that the infiltration process is significantly influenced by the top and bottom flow layers, stitching points, and yarn channels. Resin-rich zones, created by stitching, were identified as critical factors. A transparent RTM tool was developed to visually track resin flow, and microsection evaluations were used to assess the composite thickness and fiber volume content. In addition to these contributions, other recent studies [18,19] have led to advances in the optimization of composite manufacturing processes, covering improved pultrusion technology for complex geometries, and the use of data-driven/machine learning frameworks to control and optimize heterogeneous process parameters.

This Special Issue presents a diverse collection of studies that investigate the advanced properties and applications of composite materials. The manuscripts explore a range of topics, including the impact of hydrothermal aging on the damping properties of sisal mat-reinforced polyester composites [20], the integration of self-sensing capabilities in fiber-reinforced composites [21], and the flexural fatigue behavior of Kevlar-reinforced composites [22]. Together, these studies provide valuable insights into the development, characterization, and application of composite materials, underlining their potential to revolutionize various structural and functional applications. Recent advances beyond this Special Issue have also contributed to a deeper understanding of the advanced properties and functional applications of composite materials [23,24,25,26]. These studies examined the self-sensing capabilities and damage monitoring in fiber-reinforced composites, the effects of fiber hybridization and impact energy on the damping performance of natural fiber composites, and the dynamic viscoelastic response of hybrid and carbon fiber composites.

Additionally, this Special Issue collects a series of studies that use advanced simulation techniques to investigate the behavior and performance of composite materials. From finite element analysis of CFRP tendons [27] and timber beams [28] to numerical investigations of all-composite sandwich structures and natural-fiber-reinforced hybrids [29], these studies reveal the critical role of simulations in understanding and optimizing composite materials for diverse structural applications.

To conclude, as the Guest Editors of this fascinating Special Issue, we extend our gratitude to all of the authors for their valuable contributions and to the reviewers for their insightful feedback. Their collective efforts have made this Special Issue a comprehensive and impactful resource for researchers and practitioners in the composites field. We hope that the findings and innovations presented here will inspire further advancements in the development of sustainable and high-performance FRP composites. Moreover, we are excited to announce a Second Edition and look forward to showcasing continued progress and future breakthroughs in this vital and dynamic field of materials science.
